# Asperosaponin VI Alleviates Cisplatin‐Induced Liver Injury Through the Nrf2/HO‐1 Signaling Pathway

**DOI:** 10.1002/iid3.70454

**Published:** 2026-04-27

**Authors:** Han‐hua Li, Xiao‐ming Zhou, Chuan‐wei Sun, Zhi‐feng Huang, Zu‐an Liu, Hongming Lou, Huining Bian, Shao‐yi Zheng, Wen Lai, XueQing Yao

**Affiliations:** ^1^ Department of Gastrointestinal Surgery, Department of General Surgery Guangdong Provincial People's Hospital, Guangdong Academy of Medical Sciences, Southern Medical University Guangzhou Guangdong China; ^2^ Department of Burns and Wound Repair Surgery Guangdong Provincial People's Hospital, Guangdong Academy of Medical Sciences Guangzhou Guangdong China; ^3^ Department of Neurology The Affiliated Brain Hospital of Guangzhou Medical University Guangzhou Guangdong China

**Keywords:** apoptosis, asperosaponin VI, cisplatin, herbal medicine, HO‐1, inflammatory reaction, Nrf2, oxidative stress

## Abstract

**Background:**

Cisplatin (Cis) chemotherapy‐induced hepatotoxicity frequently leads to treatment interruption or dose reduction, ultimately compromising therapeutic outcomes. Asperosaponin VI (AVI), a bioactive triterpenoid saponin extracted from *Dipsacus asperoides*, has demonstrated anti‐inflammatory and antioxidant properties in various pathological conditions. However, its hepatoprotective efficacy against cisplatin‐induced hepatotoxicity and underlying molecular mechanisms remain poorly understood.

**Methods:**

This study utilized both in vitro (LO2 human hepatocytes) and in vivo (C57BL/6mice *n* = 4 per group) experimental approaches to investigate the protective effects of AVI against cisplatin‐induced acute hepatotoxicity. Cell viability was assessed using CCK‐8 assay, while hepatic injury was evaluated through histopathological examination, biochemical markers (ALT, AST, GSH), and TUNEL staining. The involvement of the nuclear factor erythroid 2‐related factor 2/heme oxygenase‐1 (Nrf2/HO‐1) signaling pathway was confirmed using Brusatol, a specific Nrf2 inhibitor.

**Results:**

AVI (400 μM in vitro; 20 mg/kg in vivo) significantly attenuated cisplatin‐induced cytotoxicity in LO2cells and reduced hepatic injury in mice. Treatment with AVI markedly decreased serum transaminase levels (ALT: 68.3 ± 30.4 vs. 358.1 ± 67.5 U/L, *p* < 0.001; AST: 129.0 ± 45.9 vs. 374.4 ± 49.5 U/L, *p* < 0.001), ameliorated oxidative stress, and suppressed inflammatory responses. AVI significantly upregulated Nrf2and HO‐1 protein expressions while downregulating pro‐inflammatory mediators (TNF‐α, IL‐1β, IL‐6) and apoptotic markers (Caspase‐1, Caspase‐3, NLRP3). Pharmacological inhibition of Nrf2 with Brusatol abolished the protective effects of AVI.

**Conclusions:**

AVI effectively protects against cisplatin‐induced hepatotoxicity through activation of the Nrf2/HO‐1 signaling pathway, suggesting its potential as a novel hepatoprotective agent in cisplatin‐based chemotherapy.

## 1 Introduction

Cancer remains one of the most formidable global health challenges, representing the second leading cause of mortality in the United States with a projected 2.1 million new diagnoses and 600,000 deaths in 2025 alone [1, 2]. Among the therapeutic armamentarium against cancer, chemotherapy continues to serve as a cornerstone of treatment. Cisplatin, a pioneering platinum‐based chemotherapeutic agent, exerts potent antineoplastic effects by interfering with DNA replication and transcription, thereby inducing apoptosis in rapidly dividing tumor cells [3, 4]. Its broad‐spectrum efficacy has established cisplatin as a first‐line treatment for various solid malignancies, including lung, gastric, colorectal, and genitourinary cancers [5, 6, 7].

Despite its remarkable therapeutic efficacy, the clinical utility of cisplatin is severely constrained by dose‐limiting toxicities, with hepatotoxicity representing a significant therapeutic challenge alongside nephrotoxicity and neurotoxicity [8]. Cisplatin‐induced hepatotoxicity, characterized by elevated serum aminotransferases, hepatocellular necrosis, and cholestasis, affects approximately 38.7% of patients receiving cisplatin‐based chemotherapy regimens [9].

The pathogenesis of cisplatin‐induced hepatotoxicity encompasses a complex interplay of molecular mechanisms. Central to this process is the excessive generation of reactive oxygen species (ROS) resulting from mitochondrial dysfunction and depletion of endogenous antioxidant defense systems, particularly the glutathione pool [10]. This oxidative imbalance precipitates a cascade of cellular damage, including membrane lipid peroxidation, protein carbonylation, and DNA strand breaks [11]. Concurrently, cisplatin triggers robust inflammatory responses through activation of nuclear factor‐κB (NF‐κB) signaling, culminating in enhanced production of pro‐inflammatory mediators including tumor necrosis factor‐α (TNF‐α), interleukin‐1β (IL‐1β), and interleukin‐6 (IL‐6) [12, 13]. The synergistic interaction between oxidative stress and inflammatory cascades ultimately drives hepatocellular death through activation of both intrinsic and extrinsic apoptotic pathways [14]. Critically, severe hepatotoxicity often necessitates dose reduction or treatment discontinuation, potentially compromising oncological outcomes and patient survival [15].

Natural products have emerged as promising sources of hepatoprotective compounds, offering potential advantages including multi‐target effects, favorable safety profiles, and reduced likelihood of drug resistance [16]. Traditional Chinese medicine (TCM) has a rich history of treating liver diseases, with numerous herbs demonstrating hepatoprotective properties in both preclinical and clinical studies [17]. Asperosaponin VI (AVI), a bioactive triterpenoid saponin isolated from *Dipsacus asperoides*, has garnered considerable attention due to its diverse pharmacological properties [18]. Recent investigations have demonstrated that AVI possesses potent anti‐inflammatory, antioxidant, anti‐apoptotic [18, 19]. In experimental models of various diseases, AVI has shown protective effects against myocardialischemia‐reperfusion injury, osteoarthritis, diabetic nephropathy, and neuroinflammation [19, 20, 21]. Mechanistically, AVI appears to exert its protective effects through modulation of multiple signaling pathways, including the nuclear factor erythroid 2‐related factor2 (Nrf2) antioxidant pathway, NF‐κB inflammatory cascade, and mitochondrial apoptotic machinery [21].

Accumulating evidence suggests that activation of the Nrf2/HO‐1 pathway represents a promising therapeutic strategy for preventing drug‐induced hepatotoxicity [22, 23]. Several natural compounds have been shown to confer hepatoprotection through Nrf2/HO‐1 activation, including sulforaphane, curcumin, and resveratrol [24, 25, 26]. However, the potential role of AVI in modulating this pathway for hepatoprotection against cisplatin‐induced injury remains unexplored.

To address these critical knowledge gaps and test our hypothesis, we conducted a comprehensive investigation using complementary in vitro and in vivo experimental approaches. Our study provides significant advances in understanding AVI‐mediated hepatoprotection, as demonstrated by the following key findings: AVI (400 μM in vitro; 20 mg/kg in vivo) effectively prevented cisplatin‐induced hepatocellular damage, evidenced by preserved viability, reduced serum transaminases, and ameliorated histopathology. Mechanistically, we establish for the first time that this protection is mediated through potentiation of the Nrf2/HO‐1 signaling pathway, with significant upregulation of both proteins. The essential role of Nrf2 was critically confirmed, as the specific inhibitor Brusatol completely abrogated AVI's protective effects. Furthermore, comprehensive analyses revealed that AVI co‐ordinately attenuated oxidative stress (restored GSH), inflammation (suppressed TNF‐α, IL‐1β, IL‐6, iNOS, Cox‐2), and apoptosis (inhibited Caspase‐1, Caspase‐3, NLRP3).

Collectively, our findings establish AVI as a promising natural hepatoprotective agent capable of effectively mitigating cisplatin‐induced liver injury through Nrf2/HO‐1 pathway activation. These discoveries not only provide compelling preclinical evidence supporting the therapeutic potential of AVI as an adjuvant in cisplatin‐based chemotherapy but also contribute fundamental insights into the molecular mechanisms underlying natural product‐mediated hepatoprotection. Our work establishes a robust foundation for future translational studies and opens new therapeutic avenues for developing Nrf2/HO‐1‐targeted interventions in drug‐induced hepatotoxicity.

## 2 Materials and Methods

### 2.1 Cell Culture

Human LO2 hepatocytes (Procell Biotechnology, China; catalog CM‐0111) were cultured in complete growth medium supplemented with 10% fetal bovine serum (FBS; Biosharp, China, catalog BL201A) and 1% penicillin‐streptomycin (Biosharp, China, catalogBL505A). Cells were maintained at 37°C in a humidified atmosphere of 5% CO₂ and 95% air.

### 2.2 Cell Viability Assay

LO2 cells were resuspended and counted in a medium containing 10% FBS, and the cell density was adjusted to 2 × 10^4^ cells/mL. The cells were then seeded into a 96‐well plate, with 100 μL of cell suspension added to each well. To prevent rapid evaporation, 200 μL of PBS was added to the surrounding wells. After 2 days of culture, non‐adherent cells were removed, and the medium was replaced with fresh medium. The cells were treated with AVI at concentrations of 12.5, 25, 50, 100, 200, 400, 800, and 1600 μM, and Cis at concentrations of 1.25, 2.5, 5, 10, 20, 40, and 80 μM, with six replicate wells for each concentration. Following 48 h of continuous culture, CCK‐8 reagent was added, and the plates were incubated in a 37°C incubator for 4 h in the dark. The absorbance at 450 nm was measured using a microplate reader, and the cell viability of each group was calculated [27].

### 2.3 Reactive Oxygen Species (ROS)

Intracellular ROS levels were measured using the ROS Detection Kit (Yuanye Biotechnology Co., Shanghai, China; catalog R32755‐200T). Following treatments, cells were washed with PBS and incubated with 10 µM DCFH‐DA in serum‐free medium at 37°C for 30 min in the dark. The DCFH‐DA is deacetylated by cellular esterases to non‐fluorescent DCFH, which is then oxidized by intracellular ROS to highly fluorescent dichlorofluorescein (DCF). After incubation, cells were washed three times with cold PBS. Fluorescence intensity was immediately measured using a microplate reader at excitation/emission wavelengths of 485/535 nm. Results are expressed as relative fluorescence intensity compared to the control group [28].

### 2.4 Mice Models of Cisplatin‐Induced Liver Damage and Tissue Harvesting

C57BL/6 male mice (*n* = 4 per group, aged 8–10 weeks) were purchased from the Guangdong Medical Laboratory Animal Center in China. The sample size was determined in adherence to the animal ethical “3R principles” and with reference to established practices in the literature [29, 30]. The mice were subjected to a 12‐h dark and light cycle under controlled conditions (25°C, 70% humidity) for 1 week, and received food and water ad libitum. The mice were then divided into six groups as follows:

i. Control: mice were administered saline as a control for five consecutive days.
ii. AVI group: mice received the same amount of AVI (20 mg/kg iv) (Yuanye Biotechnology Co., Shanghai, China; catalog A10275) for 5 consecutive days.
iii. AVI+Cis‐MOD: mice received 20 mg/kg AVI for five consecutive days and a single dose of cisplatin (30 mg/kg ip) [31].
iv. Cis‐MOD: mice were administered saline for five consecutive days, followed by a single dose of cisplatin (30 mg/kg).
v. Bru+AVI+Cis: Brusatol (Bru, Selleck, S7956) was administered intraperitoneally to the experimental mice 7 days prior, mice received AVI 20 mg/kg + Bru 0.4 mg/kg for five consecutive days and a single dose of cisplatin (30 mg/kg).
vi. DMSO + AVI+Cis: Dimethyl sulfoxide (DMSO) was administered intraperitoneally to the experimental mice 7 days prior, mice received AVI 20 mg/kg + DMSO for five consecutive days and a single dose of cisplatin (30 mg/kg).

To induce acute liver injury, all groups, except the Control and AVI groups, were intraperitoneally injected with a single dose of cisplatin (Maokang Biotechnology Co., Shanghai; catalog MZ3502) on Day 4. The mice were euthanized 24 h after cisplatin injection, and their liver and serum samples were collected for subsequent analyses [31].

In order to suppress the expression of Nrf2, Bru (0.4 mg/kg ip) was administered intraperitoneally to the experimental mice 7 days prior, while DMSO was injected as a control to assess the therapeutic effects of AVI on cisplatin‐induced liver injury. The modeling procedure and specimen acquisition have been described earlier [32]. All experimental protocols involving animals were approved by the Ethics Review Committee of Guangdong Provincial People's Hospital (No: KY‐Z‐2022‐2274‐01).

### 2.5 Gene Expression Analysis

For gene expression analysis, total RNA was extracted from each sample using a TRIzol kit (Invitrogen Life Technologies, USA; catalog 15596026) according to the manufacturer's instructions [33]. The extracted RNA (1 μg) was reverse transcribed into cDNA with Prime Script RT Enzyme mix buffer, and incubated at 37°C for 15 min, then at 85°C for 5 s. The primer sequences are shown in Table 1; β‐actin was used as an internal reference. All programs were completed using the Fast Real‐Time PCR System (Applied Biosystems, Carlsbad, CA, USA) under the following conditions: initial denaturation at 95°C for 30 s, then 40 cycles of 95°C for 5 s and 60°C for 34 s. The method used for relative quantification of gene expression was $2^{-∆∆C_{t}}$. β‐actin expression value was also normalized using this method. The primer sequences were designed using the GenBank webtool.

**Table 1:** The primers used in this study.

Goal	Primer sequence
IL‐1β	forward 5′‐GCAACTGTTCCTGAACTCAACT‐3′
	reverse 5′‐ATCTTTTGGGGTCCGTCAACT‐ 3′
TNF‐α	forward 5′‐CCCTCACACTCAGATCATCTTCT‐3′
	reverse 5′‐GCTACGACGTGGGCTACAG‐ 3′
IL‐6	forward 5′‐CCAAGAGGTGAGTGCTTCCC‐3′
	reverse 5′‐CTGTTGTTCAGACTCTCTCCCT‐ 3′
iNOS	forward 5′‐GTTCTCAGCCCAACAATACAAGA‐3′
	reverse 5′‐GTGGACGGGTCGATGTCAC‐ 3′
Cox‐2	forward 5′‐CACCCTGACATAGACAGTGAAAG‐3′
	reverse 5′‐CTGGGTCACGTTGGATGAGG‐3′
β‐actin	forward 5′‐GGCTGTATTCCCCTCCATCG‐3′
	reverse 5′‐CCAGTTGGTAACAATGCCATGT‐ 3′

### 2.6 Protein Expression Analysis

We measured protein expression profiles using western blotting. Total proteins from LO2 cells or liver tissues were lysed and extracted using RIPA buffer (Beyotime Biotechnology, Shanhai; catalog P0013B) and the cracking liquid was centrifuged at 1000*g* for 5 min to remove the tissue. The protein concentration was measured using a BCA Protein Assay kit (Thermo Scientific, Germany; catalog A55864). The protein (20 μg) was separated using 10% or 12% SDS polyacrylamide gel electrophoresis, transferred onto a polyvinylidene fluoride membrane (Millipore Corp, Germany; catalog IPVH00010), blocked with 5% non‐fat dry milk for 1 h and incubated overnight at 4°C with primary antibodies: Nrf2 (Cell Signaling Technology, Danvers, MA USA; catalog 12721), HO‐1 (Cell Signaling Technology, USA; catalog 86806), NF‐κB(Cell Signaling Technology, USA; catalog3033T) Caspase‐1(Cell Signaling Technology, USA; catalog2225S), Caspase‐3(Cell Signaling Technology, USA; catalog9662S), NLRP‐3(Cell Signaling Technology, USA; catalog15101S) and GAPDH (Cell Signaling Technology, USA; catalog 5174). The membrane was then washed with TBS‐T buffer and incubated with horseradish peroxidase (HRP)‐conjugated goat anti‐rabbit IgG (H&L) secondary antibody (Servicebio, China; catalog GB23204) at a dilution of 1:3000. The antibodies were stored at room temperature in blocking buffer at the recommended dilutions for 2 h. Western blotting was performed by washing with TBS‐T buffer three times for 5 min each [34]. Western blot images were captured using a CCD camera (Bio‐Rad ChemiDoc MP Imaging System, USA), and quantitative analysis was performed by measuring the chemiluminescent signals; band intensities were quantified as integrated optical density (IOD) using ImageJ software with local background subtraction for each band, and the net IOD of each target protein was normalized to that of the loading control (GAPDH) from the same membrane, with data presented as mean from three independent biological replicates [35].

### 2.7 Identification of Biochemical Markers

The levels of alanine aminotransferase (ALT; Nanjing Jiancheng Bioengineering Institute, China; C009‐3‐1), aspartate aminotransferase (AST; Nanjing Jiancheng Bioengineering Institute, China; C010‐3‐1) and glutathione (GSH; Nanjing Jiancheng Bioengineering Institute, China; A061‐1‐2) in serum were measured using an automated biochemical analyzer according to the manufacturer's instructions.

### 2.8 Histological Analysis

The liver tissues were fixed in 4% paraformaldehyde for 24 h. Subsequently, the samples were embedded in paraffin blocks and sectioned into 5 mm‐thick slices. Hematoxylin‐eosin (HE) staining was performed according to standard protocols. Finally, the sections were sealed, examined under a microscope, and photographed. Pathological scores were assigned based on the degree of inflammation, necrosis, congestion, and edema observed in the tissues [36]. A semi‐quantitative scoring system was employed for various pathological parameters, categorizing the severity of lesions into four grades: normal (0), mild (1), moderate (2), and severe (3). The specific scoring criteria were as follows: Firstly, focal inflammatory cell infiltration was assessed by blindly examining 20 distinct fields from the section. The lesions were graded as: 0 (normal): no foci; 1 (mild): < 2 foci per 100x field; 2 (moderate): 2‐4 foci per 100x field; and 3 (severe): > 4 foci per 100x field [37]. Secondly, hepatocyte necrosis, congestion and edema were scored based on the percentage of affected hepatocytes in the centrilobular area: 0 (normal); 1 (mild): involvement of < 10% of hepatocytes; 2 (moderate): involvement of 10%–50% of hepatocytes; and 3 (severe): involvement of > 50% of hepatocytes [38]. Apoptosis was detected using a TUNEL assay (Servicebio, China, G1502), followed by nuclear staining with DAPI. Subsequently, an anti‐fluorescence quenching reagent was applied, the slides were sealed prior to observation, and images were collected under a fluorescence microscope (Olympus, Japan).

### 2.9 Statistical Analysis

Data are presented as mean ± standard deviation. Single‐factor analysis of variance and Tukey–Kramer multiple comparison tests were used to compare the mean values among different groups. The analyses were conducted using GraphPad Prism (version 8.0, USA) and SPSS (version 24.0, USA) software. A *p*‐value less than 0.05 was considered a significant difference.

## 3 Results

### 3.1 Protective Efficacy of AVI Against Cisplatin‐Induced Cytotoxicity

**Figure 1:**
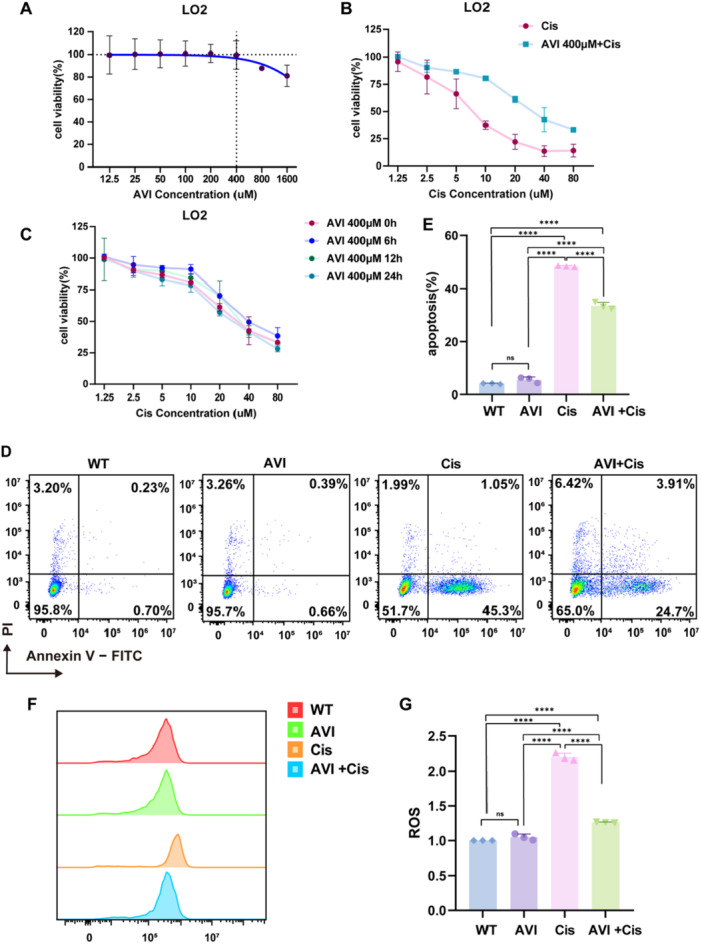
Protective efficacy of AVI against cisplatin‐induced cytotoxicity and apoptosis in LO2 cells. (A) Indicates that LO2 cell viability was not affected by AVI intervention at concentrations ranging from 0 to 400 μM over a period of 2 days. (B) Indicates a significant difference in LO2 cell viability following cisplatin treatment with vs. without AVI co‐culture. (C) Illustrates the dose‐response curves of cell viability to cisplatin following different pretreatment durations (0, 6, 12, and 24 h) with AVI. (D) Shows the apoptosis rate in LO2 cells was quantified using Annexin V‐FITC double staining, (E) shows Cis group has the highest rate of cell apoptosis, while co‐culture with AVI significantly reduced the overall cell apoptosis rate caused by Cis. (F) Shows the apoptosis of cells in each group. Apoptosis was significantly observed in the Cis group, while AVI co‐culture could significantly improve apoptosis. (G) Shows the total amount of ROS produced by cells in each group. The Cis group significantly increased ROS, while AVI co‐culture could significantly reduce the generation of ROS within cells. (*n* = 3, *p < 0.05, **p < 0.01,***p < 0.001).

To establish the safety profile of AVI in hepatocytes, we first examined its cytotoxicity in LO2 cells across a broad concentration range (0–1600 μM) over 48 h. Cell viability analysis using CCK‐8 assay revealed that AVI concentrations up to 400 μM did not significantly affect cell viability (Figure 1A). Cell viability remained above 98% at concentrations ≤ 400 μM (vs. Control, *p* > 0.05), while higher concentrations (800 and 1600 μM, *p* < 0.05) demonstrated mild cytotoxicity. Based on these findings, 400 μM was selected as the maximum non‐toxic concentration for subsequent experiments. Cisplatin exhibited concentration‐dependent cytotoxicity in LO2 cells, with significant cell death observed at concentrations ≥ 10 μM (Figure 1B). The IC50 value was determined to be 22.2 ± 6.9 μM after 48 h of treatment. For mechanistic studies, we selected 10 μM cisplatin (approximately 40% of IC50) to induce moderate, but consistent hepatocellular damage while maintaining sufficient viable cells for downstream analyses. Co‐treatment with AVI (400 μM) significantly ameliorated cisplatin‐induced cell death in a time‐dependent manner (Figure 1C). At 24 h post‐treatment, cell viability in the cisplatin group (10 μM) was 37.4% ± 4.7%, which was significantly improved to 78.4% ± 5.2% (*p* < 0.001) in the AVI co‐treatment group, demonstrating consistent cytoprotective efficacy.

### 3.2 AVI Effectively Suppresses Cisplatin‐Induced Apoptosis in LO2 Cells

Annexin V‐FITC dual staining was employed to quantify apoptosis. In the wild‐type (WT) and AVI‐only groups, early apoptosis rates were minimal (0.70% and 0.66%, respectively), with no significant difference (*p* > 0.05), confirming that AVI alone does not trigger apoptotic pathways. In contrast, cisplatin treatment markedly increased early apoptosis to 45.3%, significantly higher than both control and AVI groups (*p* < 0.001). Notably, AVI co‐treatment reduced early apoptosis to 24.7% (*p* < 0.001 vs. cisplatin alone), indicating potent anti‐apoptotic activity. Late apoptosis (Annexin V⁺/PI⁺) remained low across all groups (0.23%–3.91%), suggesting that cisplatin primarily induces early‐stage apoptosis rather than late‐stage necrosis (Figure 1D,F). The proportion of viable cells (Annexin V⁻/PI⁻) decreased from 95.8% (WT) to 51.7% (cisplatin) but was partially restored to 65.0% with AVI co‐treatment, further supporting its protective role. Consistent with these findings, cisplatin treatment induced a significant increase in intracellular reactive oxygen species (ROS), which was effectively suppressed by AVI co‐administration (Figure 1G), indicating that AVI mitigates oxidative stress—a key driver of cisplatin‐induced hepatotoxicity.

### 3.3 AVI Prevents Cisplatin‐Induced Hepatic Enzyme Elevation and Liver Damage In Vivo

**Figure 2:**
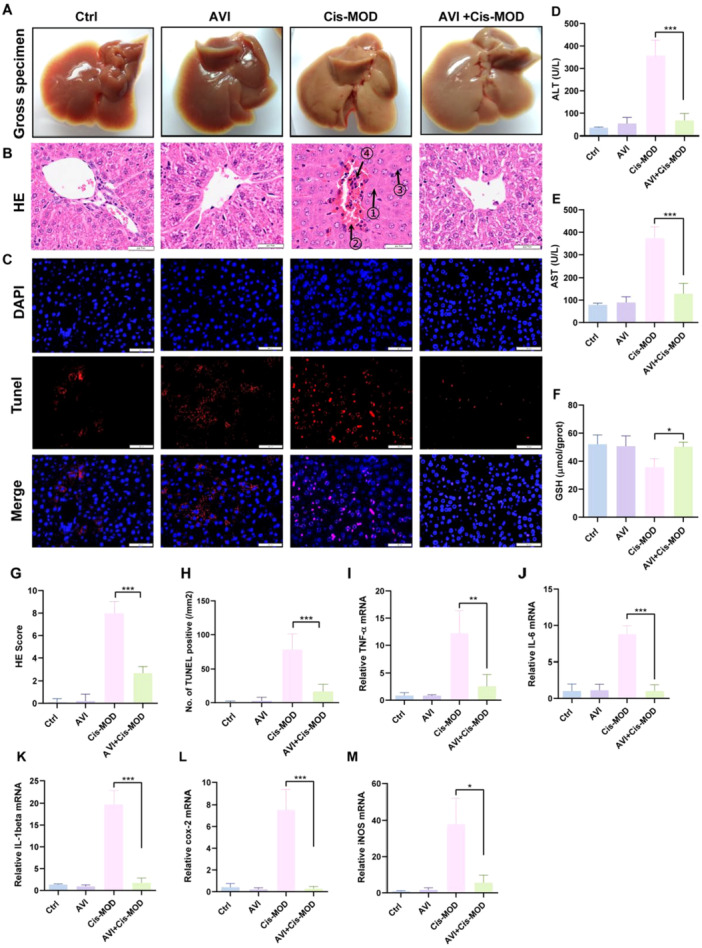
AVI Prevents cisplatin‐induced hepatic enzyme elevation and tissue damage in mice. (A) presents liver tissues from the Ctrl, AVI, Cis‐MOD, and AVI+Cis‐MOD groups, Cis‐MOD group examination of the liver tissue revealed an irregular and dull surface. (B) Presents representative H&E‐stained liver sections. Compared with the normal histoarchitecture observed in the Ctrl and AVI groups, the Cis‐MOD group displayed marked ① hepatocellular necrosis: karyolysis, and loss of cellular architecture. ② congestion: dilatation of hepatic sinusoids and central veins, packed with erythrocytes. ③ inflammatory cell infiltration: focal inflammatory cell infiltration ④ hepatocyte edema (ballooning degeneration): where hepatocytes appear swollen with pale, loss of hepatic sinusoids. All these pathological changes were markedly alleviated in the AVI+Cis‐MOD group. (C) Reveal the TUNEL staining (red fluorescence) was performed to evaluate the level of apoptosis in cells. Representative images are shown from repeated experiments. Scale bar = 50 μm. (D–F) Show the evaluated expression levels of ALT, AST, and GSH, respectively, in blood samples from the four groups (*n* = 4, *p < 0.05, **p < 0.01,***p < 0.001). (G) Presents the HE score table. (H) Show relative TUNEL⁺ cell density (cells/mm²) across the different groups (*n* = 20, 20 fields per group were analyzed from four mice per group, ***p < 0.001). (I–M) Show the TNF‐α, IL‐1β, IL‐6, iNOS, and Cox‐2 mRNA expression examined using qRT‐PCR. (*n* = 3, *p < 0.05, **p < 0.01, ***p < 0.001).

To determine the optimal hepatoprotective dose of AVI in vivo, we evaluated four doses (0, 5, 10, and 20 mg/kg) in a cisplatin‐induced liver injury model (30 mg/kg, ip). Cisplatin significantly elevated serum alanine aminotransferase (ALT) and aspartate aminotransferase (AST) levels compared to controls. AVI treatment showed dose‐dependent protection: ALT decreased from 139.1 ± 16.4 U/L (cisplatin only) to 110.4 ± 25.1 (5 mg/kg, *p* > 0.05), 89.5 ± 55.4 (10 mg/kg, *p* > 0.01), and 46.4 ± 15.5 U/L (20 mg/kg, *p* < 0.001). A similar trend was observed for AST (cisplatin: 218.7 ± 24.6 U/L; AVI 20 mg/kg: 118.5 ± 24.0 U/L, *p* < 0.001). Based on these results, 20 mg/kg was selected for further mechanistic studies. Using this optimal dose, we found that AVI pre‐treatment significantly attenuated cisplatin‐induced increases in ALT (68.3 ± 30.4 U/L, 80.9% reduction, *p* < 0.001) and AST (129.01 ± 45.88 U/L, 65.5% reduction, *p* < 0.001) (Figure 2D,E). Cisplatin also caused a marked depletion of hepatic glutathione (GSH) levels (35.7 ±  6.0 vs. 52.1 ± 6.6 μmol/g protein in control, *p* < 0.001), indicating severe oxidative stress (Figure 2F). AVI pre‐treatment preserved GSH content (50.3 ± 3.2 μmol/g protein, *p* < 0.001 vs. cisplatin), demonstrating strong antioxidant capacity.

H&E staining revealed normal hepatic architecture in control and AVI‐only groups, characterized by well‐organized hepatocytes and intact sinusoids. In contrast, cisplatin‐treated livers exhibited extensive hepatocyte necrosis, tissue edema, inflammatory infiltration, and vascular congestion (Figure 2B). The histopathological injury score (HE score) was significantly elevated in the cisplatin group (8.00 ± 1.03 vs. 0.10 ± 0.31 in control, *p* < 0.001), whereas AVI pre‐treatment dramatically reduced injury severity (2.70 ± 0.57, *p* < 0.001) (Figure 2G). TUNEL staining confirmed that cisplatin induced widespread hepatocyte apoptosis (78.34 ± 22.84 TUNEL⁺ cells/mm², *p* < 0.001 vs. control), which was significantly reduced by AVI pre‐treatment (16.89 ± 10.37 cells/mm², *p* < 0.001) (Figure 2C).

### 3.4 AVI Attenuates Cisplatin‐Induced Hepatic Inflammatory Gene Expression

Quantitative RT‐PCR analysis revealed significant upregulation of multiple inflammatory genes in cisplatin‐treated liver tissues. TNF‐α mRNA expression was increased by 14.2 ± 4.7‐fold (*p* < 0.001), IL‐1β by 15.1 ± 2.3‐fold (*p* < 0.001), and IL‐6 by 8.5 ± 1.1‐fold (*p* < 0.001) compared to control animals. AVI pre‐treatment significantly suppressed the expression of these inflammatory mediators: TNF‐α (4.7 ± 1.6‐fold, *p* < 0.001), IL‐1β (11.6 ± 4.8‐fold, *p* < 0.001), and IL‐6 (8.1 ± 1.4‐fold, *p* < 0.001) compared to cisplatin treatment alone (Figure 2I–K). Similarly, cisplatin‐induced expression of iNOS and COX‐2 was significantly attenuated by AVI (iNOS: 6.8 ± 2.5‐fold; COX‐2: 25.0 ± 6.6‐fold, both *p* < 0.001 vs. cisplatin alone) (Figure 2L,M). These findings indicate that AVI exerts potent anti‐inflammatory effects at the transcriptional level.

### 3.5 Transcriptomic Analysis of LO2 Cells Demonstrates That AVI Attenuates Cisplatin‐Induced Alterations in the Gene Expression Profile by Suppressing Apoptosis and Oxidative Stress Pathways

**Figure 3:**
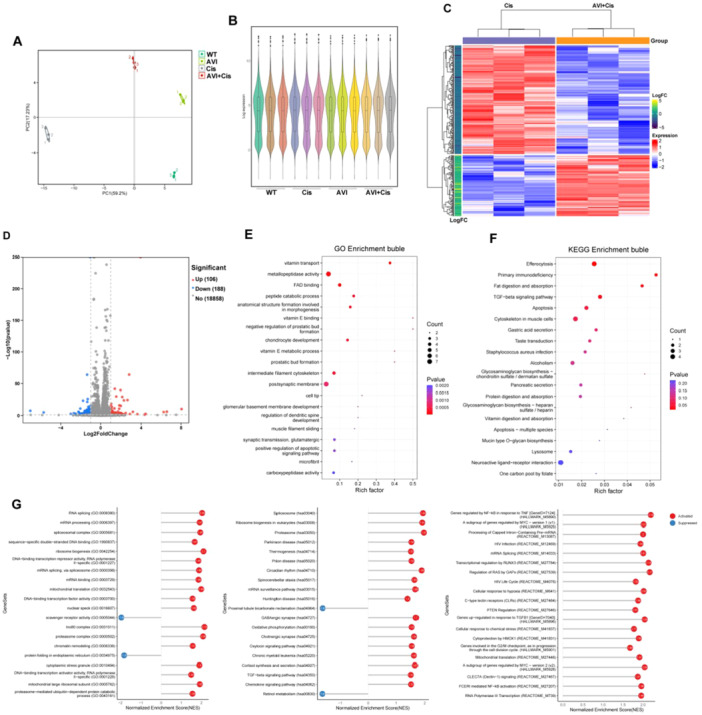
Transcriptome sequencing results from four experimental groups of LO2 cells. (A) Principal component analysis (PCA) shows clear separation along PC1 among different treatment groups, indicating value for further analysis. (B) Gene expression distribution (violin plot) demonstrates consistency in expression levels across samples. (C) Heatmap of differentially expressed gene clustering, and (D) Volcano plot of differentially expressed genes, both reveal significant differential gene expression between the Cis group and the AVI+Cis group. (E) Gene Ontology (GO) enrichment analysis shows that the gene set is significantly enriched in the three categories: biological process, molecular function, and cellular component. (F) Based on KEGG pathway enrichment analysis results, the gene set primarily covers key physiological and pathological processes such as immune defense, apoptosis, and signal transduction. (G) Results of Gene Set Enrichment Analysis (GSEA) show that gene sets involved in oxidative stress, inflammatory response, and programmed cell death are significantly negatively enriched in AVI+Cis group.

To gain system‐level insights into AVI's protective mechanisms, we performed RNA sequencing on LO2 cells from the four experimental groups: WT, AVI, Cis, and AVI+Cis. Quality control metrics confirmed high sequencing integrity (Figure 3A,B). Differential expression analysis identified numerous genes dysregulated by cisplatin, many of which were reversed upon AVI co‐treatment (Figure 3C,D). Gene Ontology (GO) enrichment analysis revealed significant upregulation of biological processes related to apoptosis, oxidative stress, and inflammatory response in the cisplatin group (Figure 3E). KEGG pathway analysis further highlighted enrichment in apoptosisand inflammatory response signaling pathways (Figure 3F). Gene Set Enrichment Analysis (GSEA) confirmed significant negative enrichment of gene sets associated with oxidative stress, inflammation, and programmed cell death in the AVI+Cis group, indicating that AVI counteracts cisplatin‐induced transcriptional reprogramming (Figure 3G). Collectively, these data demonstrate that AVI restores global gene expression toward homeostasis by suppressing key pathological pathways.

### 3.6 AVI Activates the Nrf2/HO‐1 Pathway and Inhibits Inflammatory and Apoptotic Signaling in LO2 Cells

**Figure 4:**
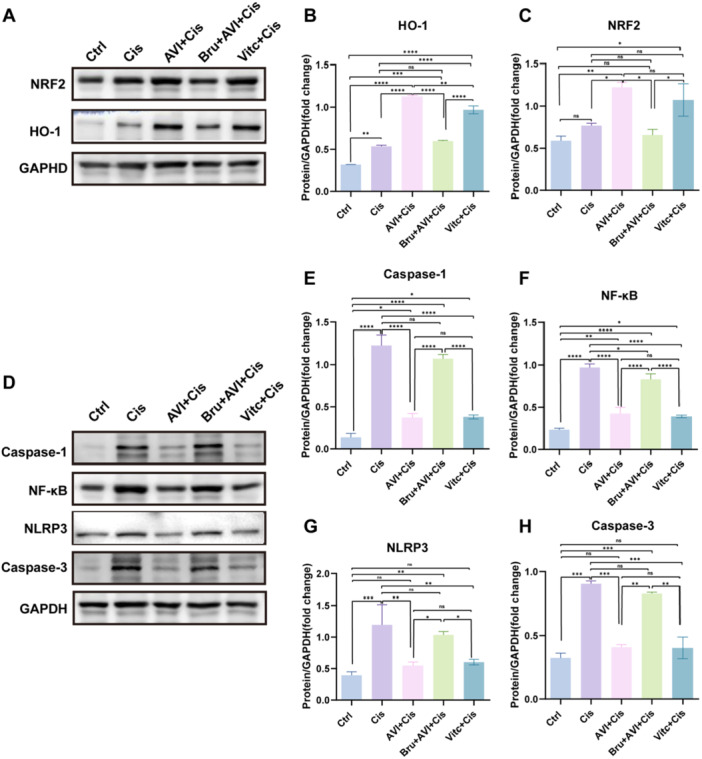
AVI suppresses hepatocyte inflammation and apoptosis in LO2 cells via activation of the Nrf2/HO‐1 axis. Total protein was extracted from the LO2cell from the control, Cis, and AVI+Cis, Bru+AVI+Cis, Vitc+Cis groups. (A–C) Representative images of western blots depicting the levels of Nrf2 and HO‐1 in the LO2 cell, and the protein/GAPDH ratios determined by densitometric analysis of the western blots. (D–H) Representative images of western blots depicting the levels of Caspase‐1, NF‐κB, NLRP3 and Caspase‐3 in the LO2 cell, and the protein/GAPDH ratios determined by densitometric analysis of the western blots.

To explore the molecular basis of AVI's cytoprotection, we examined the Nrf2/HO‐1 antioxidant pathway. Western blot analysis showed that cisplatin (10 μM, 48 h) did not significantly alter Nrf2 or HO‐1 protein levels compared to control (*p* > 0.05). In contrast, AVI (400 μM) significantly upregulated both Nrf2 (1.6 ± 0.1‐fold, *p* < 0.01) and HO‐1 (2.1 ± 0.1‐fold, *p* < 0.001) relative to cisplatin alone (Figure 4A–C). Immunofluorescence staining for Nrf2 revealed its clear nuclear accumulation in AVI‐treated cells relative to the control and cisplatin groups (see Supporting Information S1: Figure 1). Vitamin C (500 μM) was used as a positive control and partially restored Nrf2 and HO‐1 expression, though less effectively than AVI. To confirm Nrf2 dependence, cells were pre‐treated with brusatol (0.4 μM), a selective Nrf2 inhibitor. Brusatol abolished AVI‐induced upregulation of Nrf2 and HO‐1, confirming pathway specificity. Cisplatin significantly activated inflammatory signaling, increasing protein levels of NF‐κB, NLRP3, and Caspase‐1 (*p* < 0.001) (Figure 4D–G). AVI co‐treatment suppressed these increases: NF‐κB (2.3 ± 0.1‐fold), NLRP3 (2.1 ± 0.6‐fold), and Caspase‐1 (3.2 ± 0.3‐fold), all *p* < 0.001 vs. Cisplatin alone. Similarly, cisplatininduced Caspase‐3 activation (2.8 ± 0.2‐fold, *p* < 0.001) was significantly attenuated by AVI (2.2 ± 0.1‐fold, *p* < 0.001) (Figure 4H). Notably, brusatol reversed the anti‐inflammatory and anti‐apoptotic effects of AVI, leading to elevated expression of all markers (*p* < 0.001), confirming that AVI's protective effects are mediated through Nrf2 activation.

### 3.7 AVI‐Mediated Hepatoprotection Requires Nrf2/HO‐1 Signaling Activation

**Figure 5:**
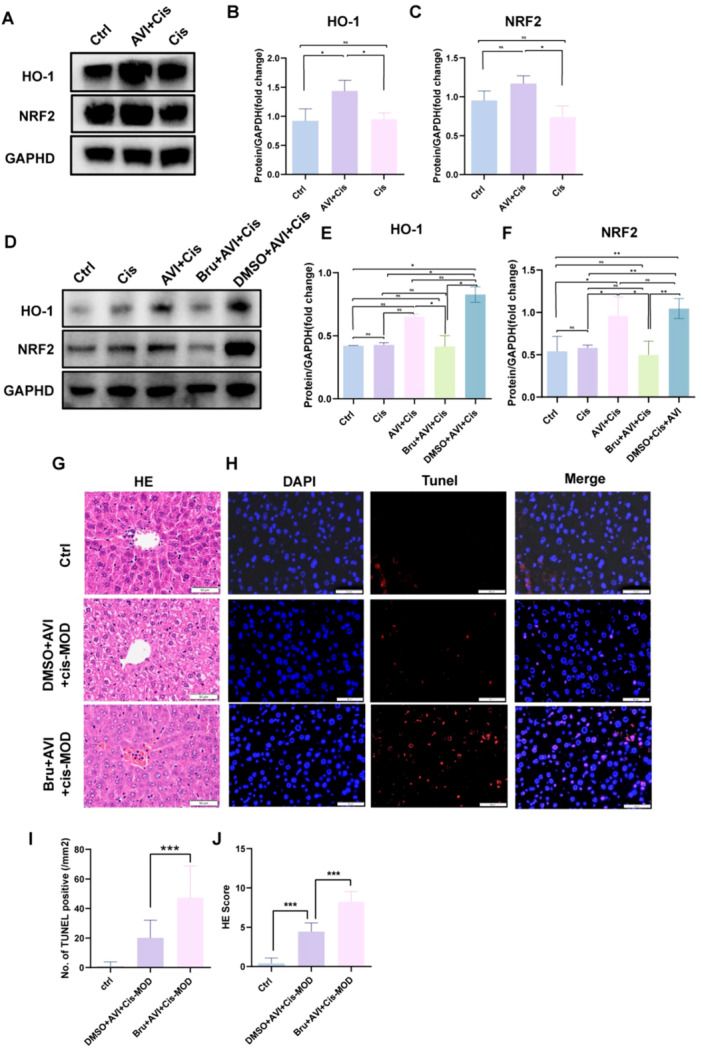
Total protein was extracted from the liver tissues from the control, Cis‐MOD, and AVI+Cis‐MOD groups. (A–C) Representative images of western blots depicting the levels of Nrf2 and HO‐1 in the liver, and the protein/GAPDH ratios determined by densitometric analysis of the western blots. (D–F) Representative images of western blots depicting the levels of Nrf2 and HO‐1 in the liver after the administration of an Nrf2 inhibitor. as well as the protein/GAPDH ratios determined through densitometric analysis. (H, I) TUNEL staining (red fluorescence) was performed to evaluate the level of apoptosis in cells after the administration of an Nrf2 inhibitor. Representative images are shown from repeated experiments. Scale bar = 50 μm. (G and J) Illustrates the comprehensive scores of cell edema, Congestion, necrosis, and inflammation across the three groups. (*n* = 20, ***p < 0.001).

Western blot analysis confirmed that cisplatin did not significantly alter hepatic Nrf2 or HO‐1 expression (*p* > 0.05 vs. control). However, AVI pre‐treatment significantly increased both proteins (Nrf2: 1.6 ± 0.1‐fold; HO‐1: 1.5 ± 0.2‐fold, *p* < 0.001) (Figure 5A–C). To validate the functional role of Nrf2 in vivo, mice were pre‐treated with brusatol (0.4 mg/kg, ip, daily for 7 days). Brusatol significantly attenuated AVI‐induced upregulation of Nrf2 (0.5 ± 0.2‐fold, *p* < 0.001) and HO‐1 (0.6 ± 0.1‐fold, *p* < 0.001) (Figure 5D–F). TUNEL staining revealed that brusatol increased hepatocyte apoptosis in AVI‐treated mice (47.4 ± 21.4 vs. 20.2 ± 11.9 TUNEL⁺ cells/mm² in AVI + cisplatin group, *p* < 0.001) (Figure 5H,I). Histopathological scoring showed significantly worse injury in the brusatol group (8.25 ± 1.29 vs. 4.45 ± 1.10, *p* < 0.001) (Figure 5G,J), demonstrating that Nrf2 activation is indispensable for AVI's hepatoprotective effects.

## 4 Discussion

This comprehensive investigation provides the systematic evidence that Asperosaponin VI (AVI) confers robust hepatoprotection against cisplatin‐induced liver injury through activation of the Nrf2/HO‐1 signaling pathway. our findings demonstrate several key mediators involved in the cisplatin induced hepatotoxicity and the role of AVI in modulating these mediators. First, we established that AVI exhibits concentration‐dependent cytoprotective effects in human hepatocytes (LO2) with an optimal protective concentration of 400 μM, achieving a 31% improvement in cell survival under cisplatin stress conditions. Second, our mechanistic investigations revealed that AVI treatment resulted in a 1.6‐fold upregulation of Nrf2 expression and a 1.5‐ to 2.1‐fold increase in HO‐1 levels compared to cisplatin treatment alone. Third, the translational significance of these findings was validated in vivo, where AVI treatment (20 mg/kg) achieved clinically meaningful reductions in hepatic injury markers, with ALT levels decreasing by 80.9% and AST by 65.5% compared to cisplatin monotherapy. Most importantly, pharmacological inhibition studies using Brusatol definitively established the Nrf2‐dependence of AVI‐mediated protection, providing mechanistic certainty that distinguishes this work from previous correlative studies [39].

The Nrf2/HO‐1 pathway represents one of the most evolutionarily conserved cellular defense mechanisms against oxidative stress and xenobiotic toxicity [40]. Our findings reveal that AVI acts as a potent Nrf2 activator through mechanisms that appear to involve both direct and indirect pathways. The robust nuclear translocation of Nrf2 observed in our studies (1.6‐fold increase in hepatic tissues) suggests that AVI may function as an electrophilic compound capable of modifying critical cysteine residues in Keap1, thereby disrupting the Keap1‐Nrf2 interaction and promoting Nrf2 stabilization [41]. The substantial upregulation of HO‐1 expression (1.5‐fold in liver tissues) observed in our study represents a critical mediator of AVI's protective effects. HO‐1 catalyzes the rate‐limiting step in heme degradation, producing three bioactive molecules: biliverdin (subsequently reduced to bilirubin), carbon monoxide (CO), and free iron [42]. Each of these products contributes to cytoprotection through distinct mechanisms: bilirubin functions as a potent lipophilic antioxidant capable of scavenging peroxyl radicals and inhibiting lipid peroxidation [43]; CO acts as a gaseous signaling molecule that modulates inflammatory responses and prevents apoptosis through activation of soluble guanylate cyclase and p38 MAPK pathways [44]; and the transient increase in free iron, while potentially pro‐oxidant, ultimately leads to adaptive upregulation of ferritin and other antioxidant systems [45].

Our correlation analyses demonstrate significant inverse relationships between Nrf2 expression and inflammatory gene expression (TNF‐α, IL‐1β, IL‐6), indicating that AVI‐mediated Nrf2 activation drives a potent anti‐inflammatory response. This finding aligns with recent mechanistic studies demonstrating that Nrf2 can directly suppress NF‐κB signaling through multiple mechanisms, including competition for transcriptional co‐activators, direct protein‐protein interactions, and epigenetic modifications of inflammatory gene promoters [46, 47]. The 54% reduction in NF‐κB expression in AVI‐treated cells (vs. Cis alone) demonstrates functional crosstalk between the pathways, which may represent a key mechanism underlying the compound's dual antioxidant and anti‐inflammatory properties.

In the presence of infectious agents or cellular stress signals, these molecular entities undergo structural reorganization through oligomerization and assembly, creating a functional platform that orchestrates both inflammatory initiation and NLRP3 inflammasome assembly, consequently resulting in caspase‐1 activation, which hydrolyzes pro‐IL‐1β into IL‐1β [48]. IL‐1β induces the release of pro‐inflammatory cytokines, including TNF‐α, IL‐8, and IL‐6, leading to the amplification of the inflammatory cascade [49, 50]. IL‐1β is a classic inflammation‐inducing cytokine that up‐regulates inflammatory mediators such as NO and PGE2 by regulating iNOS and Cox‐2 targets [51]. iNOS belongs to the NOS family and is responsible for NO synthesis. Overproduction of iNOS leads to O2 and NO production, and subsequently forms ONOO‐, triggering biomolecular damage and apoptosis [52]. Another important inflammatory factor, Cox‐2, regulates PGE2 production and further activates the PKCδ/Pyk2/c‐Src/EGFR/PI3K/Akt pathway, which is crucial for airway inflammation generation [53]. The results of this study demonstrated that AVI significantly suppressed cisplatin‐induced increases in ALT/AST, mitigated inflammation and oxidative stress, downregulated key cytokines (IL‐1β, IL‐6, COX‐2, iNOS, TNF‐α), and attenuated hepatocyte apoptosis in mice.

Several natural compounds have been reported to activate Nrf2 signaling and provide hepatoprotection, including sulforaphane, curcumin, and quercetin [54]. However, AVI demonstrates several unique advantages in the context of cisplatin‐induced hepatotoxicity. Sulforaphane, while a potent Nrf2 activator, has limited bioavailability and stability issues that restrict its therapeutic application [55]. Curcumin, despite extensive research, suffers from poor aqueous solubility and rapid metabolism that necessitates complex delivery systems [56]. Quercetin is a well‐established and potent Nrf2 agonist. Its primary limitations are poor aqueous solubility and consequent low oral bioavailability, which significantly hinder its translational potential as a therapeutic agent [57]. In contrast, AVI demonstrated consistent protective effects at achievable concentrations both in vitro (400 μM) and in vivo (20 mg/kg), suggesting favorable pharmacokinetic properties that warrant further investigation.

This study has certain limitations. First, the sample size in the animal experiments was relatively small, which may limit the statistical power and generalizability of the therapeutic outcomes. Second, and more importantly, our findings are based on an acute, high‐dose cisplatin challenge model. While this model is valuable for elucidating primary injury mechanisms and evaluating immediate protective effects, it does not fully recapitulate the chronic or repeated low‐dose exposure regimens typical of clinical chemotherapy. Consequently, the longitudinal persistence of the therapeutic benefits observed here requires further validation through extended studies that employ more clinically relevant dosing schedules.

## 5 Conclusions

**Figure 6:**
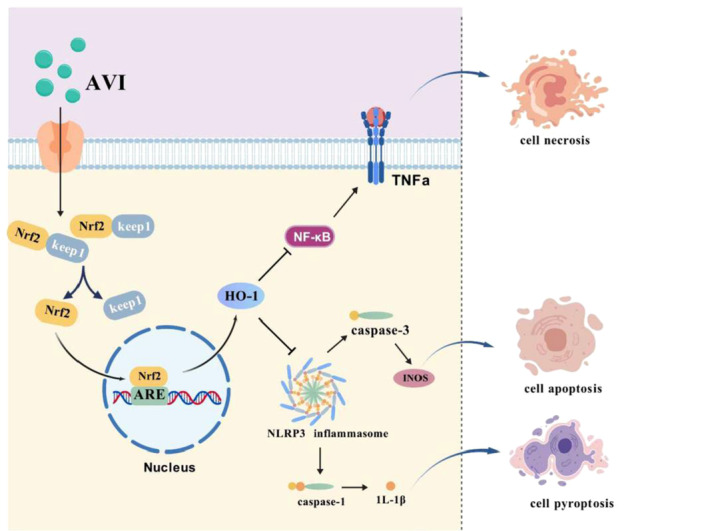
AVI alleviates cisplatin‐induced hepatic injury, potentially via the Nrf2/HO‐1 signaling axis.

The experimental results demonstrate that AVI ameliorates cisplatin‐induced oxidative stress and inflammatory responses through activation of the nuclear factor erythroid 2‐related factor 2/heme oxygenase‐1 (Nrf2/HO‐1) signaling axis, as illustrated in Figure 6. Based on these findings, we recommend further studies to explore the therapeutic potential of AVI for alleviating chronic liver injury and systemic oxidative damage associated with cisplatin‐based chemotherapy.

## Author Contributions

**Han‐hua Li:** conceptualization, methodology, software, writing – original draft, and data curation. **Xiao‐ming Zhou:** software, writing – original draft, conceptualization, and data curation. **Chuan‐wei Sun:** formal analysis, data curation, writing – original draft, and software. **Zhi‐feng Huang:** software, formal analysis, writing – original draft, and methodology. **Zu‐an Liu:** investigation and funding acquisition. **Hongming Lou:** methodology, formal analysis, and software. **Huining Bian:** validation and visualization. **Shao‐yi Zheng:** writing – review and editing. **Wen Lai:** project administration, resources, writing – review and editing. **XueQing Yao:** funding acquisition, writing – review and editing, and supervision.

## Conflicts of Interest

The authors declare no conflicts of interest.

## Supporting information

Supporting Figure

## Data Availability

The data that support the findings of this study are available in the supporting material of this article.
