# Supplementary material for: Asperosaponin VI Alleviates Cisplatin‐Induced Liver Injury Through the Nrf2/HO‐1 Signaling Pathway
Source: Immun Inflamm Dis. 2026 Apr 27;14(4):e70454. doi: 10.1002/iid3.70454 (PMC13121569; doi:10.1002/iid3.70454)

Figurementary figure 1: Immunofluorescence staining for Nrf2 revealed its clear nuclear accumulation in AVI-treated cells.


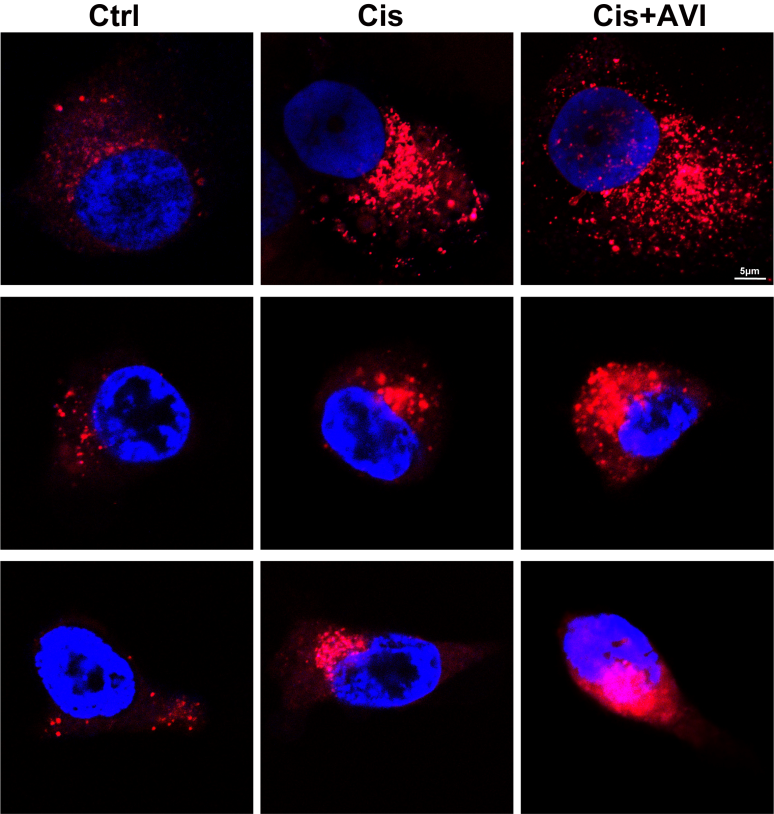

Supplement: Supplementary file 1 — Supporting Figure [file IID3-14-e70454-s001.docx]
